# Metformin prevents cell tumorigenesis through autophagy-related cell death

**DOI:** 10.1038/s41598-018-37247-6

**Published:** 2019-01-11

**Authors:** Mauro De Santi, Giulia Baldelli, Aurora Diotallevi, Luca Galluzzi, Giuditta Fiorella Schiavano, Giorgio Brandi

**Affiliations:** 1 Department of Biomolecular Sciences, Hygiene Unit, University of Urbino Carlo Bo, Urbino (PU), Italy; 2 Department of Biomolecular Sciences, Biotechnology Unit, University of Urbino Carlo Bo, Urbino (PU), Italy

## Abstract

Autophagy is a cellular mechanism by which cells degrade intracellular components in lysosomes, maintaining cellular homeostasis. It has been hypothesized that autophagy could have a role in cancer prevention through the elimination of damaged proteins and organelles; this could explain epidemiological evidence showing the chemopreventive properties of the autophagy-inducer metformin. In this study, we analyzed the autophagy-related effect of metformin in both cancer initiation and progression in non-tumorigenic cells. We also analyzed the induction of tumorigenesis in autophagy-deficient cells, and its correlation with the ER stress. Our results showed that metformin induced massive cell death in preneoplastic JB6 Cl 41-5a cells treated with tumor promoter (phorbol) and in NIH/3T3 treated with H_2_O_2_. Inhibiting autophagy with wortmannin or ATG7 silencing, the effect of metformin decreased, indicating an autophagy-related cytotoxic activity under stress conditions. We also found an induction of tumorigenesis in ATG7-silenced NIH/3T3 cell clone (3T3-619C3 cells), but not in wild-type and in scrambled transfected cells, and an upregulation of unfolded protein response (UPR) markers in 3T3-619C3 cells treated with H_2_O_2_. These findings suggest that autophagic cell death could be considered as a new mechanism by which eliminate damaged cells, representing an attractive strategy to eliminate potential tumorigenic cells.

## Introduction

Tumorigenesis is a complex and multistage process characterized by an accumulation of cellular damage promoted by chronic inflammation and exposure to carcinogens. Cancer prevention strategies could be addressed to different steps of tumorigenic process, making the organism more resistant to mutagens/carcinogens and/or to inhibit disease progression by administering chemopreventive agents, inhibiting initiation and/or progression of cell transformation^1^.

Autophagy is the cellular mechanism appointed to the degradation of cytoplasmic components, maintaining cellular homeostasis through elimination of damaged proteins and organelles. Despite autophagy is considered a survival mechanism for cancerous cells in the hostile tumor microenvironment, it could prevent chronic tissue stress that can induce cellular damage to proteins, organelles and DNA, inhibiting cancer initiation and progression^2–6^.

Metformin, one of most widely prescribed oral hypoglycemic agents, has recently received increased attention because of its potential antitumorigenic effects and because of the appealing strategy to repurpose drugs with well described safety profiles^7–11^. Several epidemiological studies have documented a correlation between metformin and reduced cancer incidence and mortality; however, both animal and epidemiological studies have shown somewhat mixed effects and the epidemiological literature relates preferentially to individuals with diabetes^12^. The chemopreventive effect of metformin in non-diabetic subjects is still to be demonstrated, and the related cellular and molecular mechanisms are largely unknown. It has been hypothesized that metformin may have anticancer properties through different mechanisms, independent of its hypoglycemic effect; its main proposed anticancer molecular action is associated with the inhibition of mTORC1 - which is involved in metabolism, growth and differentiation of cancer cells^13^ - mediated by AMPK activation or in a AMPK-independent manner. Other proposed mechanisms through which metformin could exert its anticancer effects include the induction of cell cycle arrest and/or apoptosis and the inhibition of the unfolded protein response (UPR)^14^.

The UPR includes signal transduction pathways activated to overcome the perturbations of the endoplasmic reticulum (ER) homeostasis, known as “ER stress”^15^, which is induced by an accumulation of unfolded/misfolded proteins, caused by depletion of Ca^2+^ levels, oxidative stress, low oxygen levels (hypoxia) or glucose deprivation^16^. Since the nutrient requirement of solid tumors can exceed the capacity of the cells’ microenvironment, hypoxia and glucose deprivation can occur, activating the UPR; this process is thought to be able to protect tumor cells from the stressful conditions of glucose deprivation and hypoxia as well as from immune surveillance^17^.

The crosstalk between autophagy and ER stress is well known, and these two systems are dynamically interconnected, either stimulating or inhibiting each other. Moreover, the concurrence between ER stress and autophagy is common in several human pathologies, including neurodegenerative disorders, diabetes and cancer^18^.

The aim of this study was to corroborate the role of autophagy in cancer initiation and progression, and to analyze the molecular pathways related to ER stress. Tumorigenesis was analyzed in the preneoplastic JB6 Cl 41-5a cells after autophagy inhibition with wortmannin, and in ATG7-silenced cell clones generated from non-tumorigenic NIH/3T3 cells. The autophagy-related activity of metformin in these cell models was also evaluated.

## Results

### Metformin inhibits tumor promotion through autophagy-related cell death

**Figure 1:**
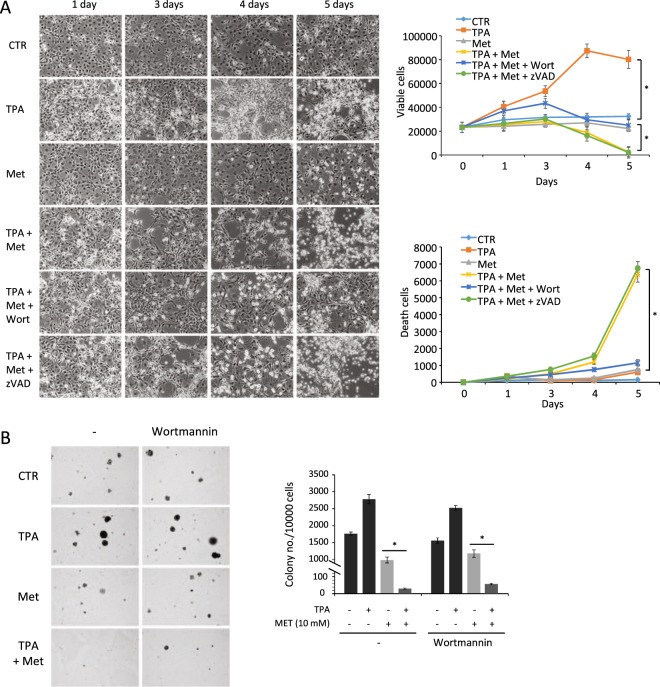
Effect of metformin on tumor promotion in JB6 P+ cells. Tumor promotion was induced with TPA and analyzed by (**A**) anchorage-dependent (adherent cells) and (**B**) –independent (soft agar) growth assays. JB6 cells were treated with TPA (10 ng/ml) and metformin (10 mM) for 5 days (adherent cells) or 3 weeks (soft agar); autophagy was inhibited with wortmannin (2 µM). Cell count of adherent cells and colony count of soft agar assays are shown as means ± SEM; *N* = 3, *P* < 0.001.

To analyze the role of autophagy in cancer promotion, the pre-neoplastic JB6 P+ cell line has been used. These cells are sensitive to the growth induction by 12-O-Tetradecanoylphorbol 13-acetate (TPA) in both anchorage-dependent and -independent culture conditions, as shown in Fig. 1. Cell count of adherent cells after 5 days of incubation with the tumor promoter TPA increased of about 2.5 fold, whereas the anchorage-independent colony formation in soft agar, a hallmark of malignant transformation, after 21 days of incubation with TPA increased of 1.6 fold. To induce autophagy during tumor promotion we treated cells with 10 mM of metformin, an hypoglycemic drug with a well note ability to induce autophagy^14^. We found that metformin neither inhibits cell viability of non-proliferating pre-neoplastic cells plated at confluence, nor colony formation in soft agar. On the other hand, in TPA-treated JB6 P+ cells, metformin showed a marked cytotoxic effect, significantly reducing the number of viable cells and increasing the number of death cells in anchorage-dependent conditions (Fig. 1A); moreover, in presence of TPA, metformin completely inhibited the colony formation in soft agar (Fig. 1B). To evaluate the role of autophagy in the cytotoxic activity of metformin, JB6 P+ cells were treated with the autophagy inhibitor wortmannin followed by TPA, metformin and combination treatments. Wortmannin alone did not significantly change cell proliferation or colony formation (not shown). Interestingly, wortmannin reversed the effect of metformin in adherent cells inhibiting its cytotoxic effect in presence of TPA (Fig. 1A), suggesting that metformin could induce autophagic cell death during tumor promotion. The effect of wortmannin was only partially confirmed in soft agar culture (Fig. 1B). To evaluate if apoptosis is involved in metformin-induced cell death, cells were treated with the pancaspase inhibitor zVAD-fmk; as shown in Fig. 1A, cytotoxic activity of metformin in TPA-stimulated cells did not change after zVAD-fmk treatment, suggesting that apoptosis is not involved in JB6 P+ cell death.

**Figure 2:**
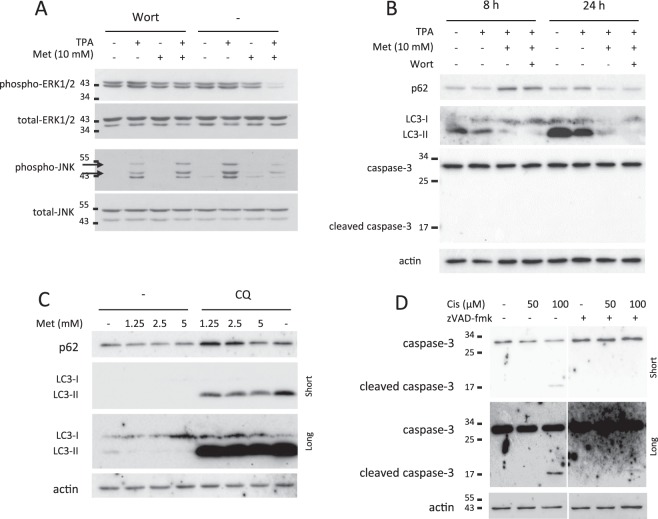
Western blotting analysis in JB6 P+ cells. Cells were cultured with TPA (10 ng/ml) for 24 hours and treated with metformin (10 mM) for additional 8 (**B**) and 24 (**A**,**B**) hours. Autophagy was inhibited with wortmannin (2 µM) (**A**,**B**) or chloroquine (100 µM) (**C**); apoptosis was induced with cisplatin (50 and 100 µM) and inhibited with zVAD-fmk (10 µM) (**D**). Phosphorylation of ERK1/2 and JNK (**A**), p62 and LC3 (**B**,**C**), and caspase-3 cleavage (**B**,**D**) were analyzed. Total ERK1/2, total JNK and actin were used as loading control. *N* = 3. Cropped blots are shown. Uncropped blots are presented in Supplementary Fig. S1.

Next, we analyzed the molecular pathways involved in metformin activity finding an inhibition of phospho-ERK1/2 in cells treated with TPA and metformin, without wortmannin; phospho-JNK was found to be stimulated in TPA-treated cells but not in presence of metformin without wortmannin (Fig. 2A). These results suggest an autophagy-related inhibition of ERK1/2 and JNK signaling by metformin. The analysis of the autophagic markers LC3 and p62 confirmed that metformin is able to induce autophagy; in fact, p62 is upregulated after 8 h and decreases after 24 hours after metformin treatment; the analysis of LC3 also showed an increase of autophagic flux induced by metformin, that is converted in wortmannin-treated cells after 24 hours (Fig. 2B). Our results also shows that metformin counteracts the activity of chloroquine, an inhibitor of the autophagic flux able to block the autophagosome fusion with lysosome; as shown in Fig. 2C, metformin reduces the accumulation of p62 and LC3-II induced by chloroquine, indicating an induction of the autophagic flux. Again, the western blotting results did not reveal caspase-3 cleavage (Fig. 2B), suggesting that the apoptotic process was not involved in the cytotoxic activity of metformin. As positive control of apoptosis, JB6 P+ cells were treated with cisplatin for 48 hours showing a dose-dependent caspase-3 cleavage (Fig. 2D); the cleavage of caspase-3 was inhibited treating cells with zVAD-fmk (Fig. 2D), confirming the efficiency of zVAD-fmk in inhibiting apoptosis in our cell model (Fig. 1A).

### Induction of tumorigenesis in shATG7 NIH/3T3 cells

**Figure 3:**
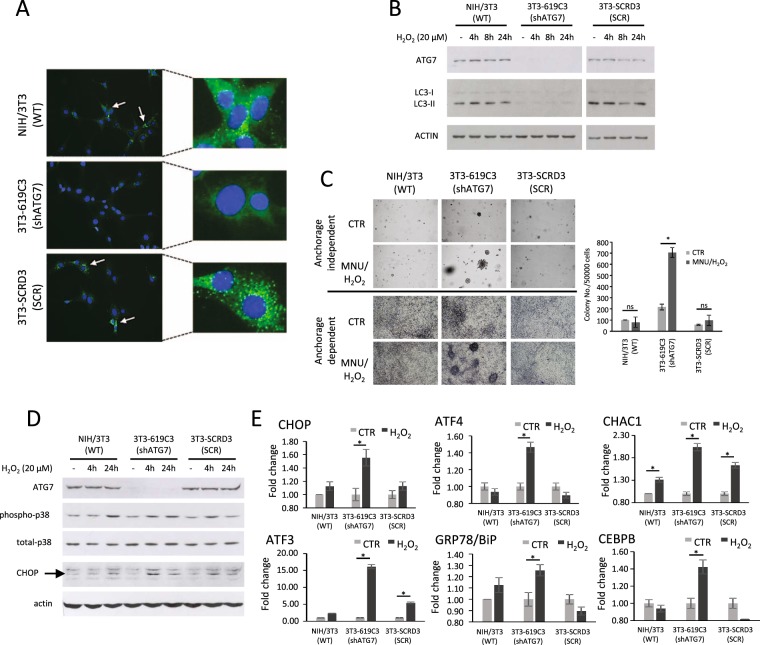
Induction of tumorigenesis in wild type, shATG7 and scrambled transfected NIH/3T3 cells, and unfolded protein response analysis. (**A**) Immunofluorescence analysis of LC3A/B protein in cells starved with serum-deprived medium for 4 hours. LC3A/B fluorescent puncta indicate autophagosome formation. (**B**) Western blotting analysis of ATG7 and LC3B-I/II in cells treated with H_2_O_2_ 20 µM for 4, 8 and 24 hours. Actin was used as loading control. *N* = 3. Cropped blots are shown. Uncropped blots are presented in Supplementary Fig. S2. (**C**) Two-stage tumorigenesis induction. Cells were treated for 1 hour with MNU (50 µg/ml) and cultured up to three weeks in medium containing H_2_O_2_ (10 µM). Cell transformation was analyzed by anchorage-dependent (adherent cells) and –independent (soft agar) growth assays. Colonies were counted after 21 days. Data are means ± SEM; *N* = 3, *P* < 0.001. (**D**) Western blotting analysis of ATG7, phospho-p38 and CHOP in cells treated with H_2_O_2_ (20 µM) for 4 and 24 hours. Actin and total p38 were used as loading control. *N* = 3. Cropped blots are shown. Uncropped blots are presented in Supplementary Fig. S2. (**E**) Real-time PCR gene expression analysis of CHOP, ATF3, ATF4, GRP78/BiP, CHAC1 and CEBPB in cells treated with H_2_O_2_ (20 µM) for 4 hours. Data are means ± SD. *N* = 3, *P* < 0.01.

To evaluate the role of autophagy in the tumorigenic cell transformation process, NIH/3T3 *Atg7*-silenced single cell clones have been generated. Cells were transfected with shRNA plasmids containing four different sequences targeted to the *Atg7* gene, that encodes an E1-like activating enzyme essential for autophagosome formation (Supplementary Materials, Table S1). Single cell clones were selected and analyzed for ATG7 protein expression as described in material and methods section. The ATG7 expression and basal lipidated LC3 amount were analyzed by western blotting to evaluate the stability of transfection (not shown). The NIH/3T3 shATG7 clone 619-C3 (3T3-619C3 hereafter) was selected for further experiments. As transfection control, the clone 3T3-SCRD3 transfected with non-effective scrambled shRNA was selected. To evaluate whether the *Atg7* silencing inhibits the autophagic process, NIH/3T3, 3T3-619C3 and 3T3-SCRD3 cells were starved in serum-deprived culture medium for 4 hours and autophagosome formation was analyzed by immunofluorescence of LC3. The results showed LC3 puncta in wild type and scrambled-transfected 3T3-SCRD3 cells, but not in ATG7-silenced 3T3-619C3 cells (Fig. 3A).

The two-stage transformation assay was carried out using MNU as tumor initiator and H_2_O_2_ as tumor initiator/promoter. First, the ATG7 expression and LC3-I/II amount were evaluated in cells treated with sub-lethal doses of H_2_O_2_. Our results showed that the ATG7 protein was not expressed in 3T3-619C3 cells, and that the level of protein did not change after H_2_O_2_ treatment (Fig. 3B). Likewise, 3T3-619C3 cells showed low amount of LC3-II protein (the lipidated form of LC3) (Fig. 3B) in both control cells and H_2_O_2_-treated cells, demonstrating that the ATG7 protein is required for the formation of autophagosomes, and sub-lethal doses of H_2_O_2_ did not induce autophagy. Next, MNU/H_2_O_2_ two-stage cell transformation was carried out and, after two weeks of treatment, cells transformation was monitored by colony formation in soft agar. Results showed that the capability of anchorage-independent proliferation significantly increased only in ATG7-silenced cells (Fig. 3C; *p* < 0.01). Moreover, morphological cell transformation was revealed by foci formation of MNU/H_2_O_2_-treated shATG7 cells in anchorage-dependent culture conditions (Fig. 3C).

There are growing evidence that the endoplasmic reticulum stress and the related UPR could have a role in cancer^19^. The UPR was investigated in NIH/3T3, 3T3-619C3 and 3T3-SCRD3 treated with H_2_O_2_ in order to evaluate the crosstalk between autophagy and the response to the endoplasmic reticulum stress. The stress marker phospho-p38 and UPR marker CHOP were analyzed by western blotting in cells treated with H_2_O_2_ (20 µM) for 4 and 24 hours (Fig. 3D). The UPR markers CHOP, ATF3, ATF4, GRP78/BiP, CHAC1 and CEBPB were also analyzed by qPCR in cells treated with H_2_O_2_ (20 µM) for 4 hours (Fig. 3E). Results showed that H_2_O_2_ treatment induced the stress marker phospho-p38 in all tested cells; instead, UPR markers were significantly upregulated more in shATG7 3T3-619C3 cells than in the wild type and/or scrambled-transfected cells treated with H_2_O_2_ (Fig. 3D,E).

### Metformin induces autophagy-related cell death in tumor-initiated cells

**Figure 4:**
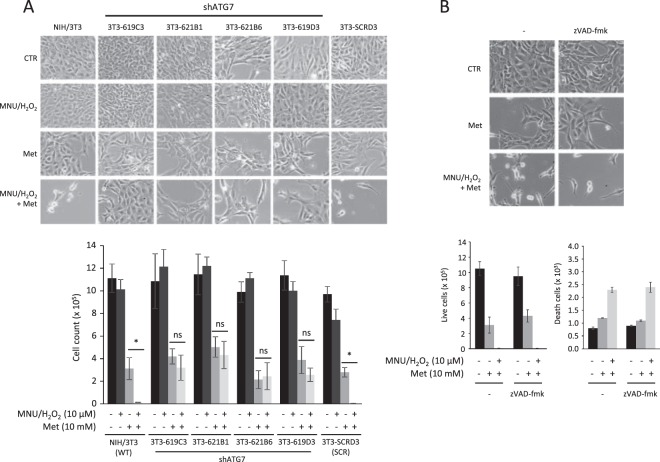
Metformin activity in wild type  (**A**), shATG7 (**A**) and scrambled transfected (**A**,**B**) NIH/3T3 cells. Cell viability were evaluated after 1 hour treatment with MNU (50 µg/ml) and 1 week culture with H_2_O_2_ (10 µM) and metformin (10 mM) (**A**,**B**), with or without zVAD-fmk (**B**). After treatments, cells were counted and photographed at 10× magnification. Data are means ± SEM; *N* = 3, *P* < 0.001.

Cell damage accumulation could predispose cells to the tumorigenic transformation. To evaluate the potential role of autophagy in inhibiting cell viability of damaged cells, NIH/3T3, 3T3-619C3, 3T3-619D3, 3T3-621B1, 3T3-621B6 and 3T3-SCRD3 cells were treated with 50 µg/ml of MNU for 1 hour and cultured for one week with 10 µM H_2_O_2_ - to induce tumorigenesis - and 10 mM metformin to induce autophagy. Cell survival was evaluated by cell count, revealing that metformin induced cytostatic effect in all cell lines; on the other hand, in MNU/H_2_O_2_ treated cells, metformin inhibited cell viability in NIH/3T3 and 3T3-SCRD3 but not in shATG7 cells (Fig. 4A), suggesting an induction of autophagy-related cell death in damaged cells.

To evaluate if apoptosis is involved in metformin-treated 3T3-SCRD3 cell death, cells were treated with metformin and MNU/H_2_O_2_ with or without the pan-caspase inhibitor zVAD-fmk. Our results show that neither cell viability nor cell death induced by metformin significantly changed in 3T3-SCRD3 cells treated with zVAD-fmk (Fig. 4B).

**Figure 5:**
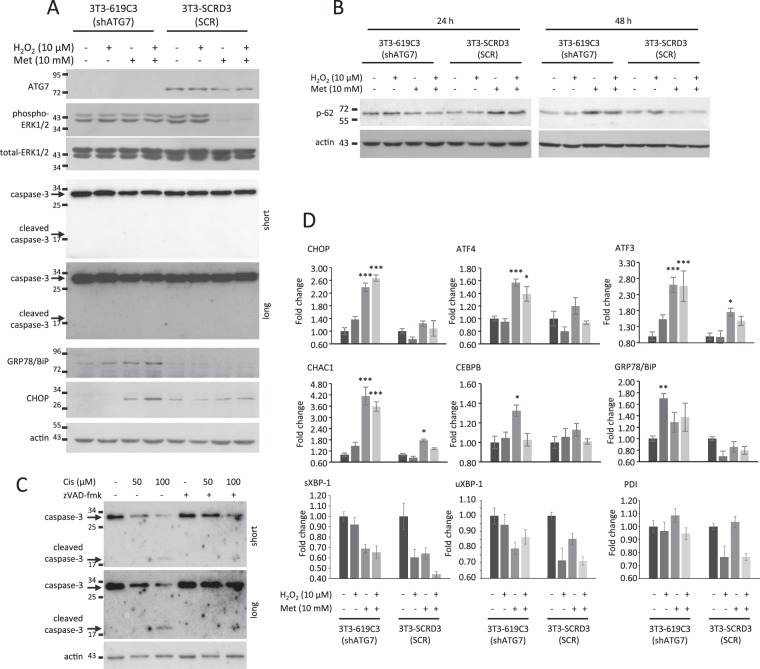
Evaluation of molecular changes in 3T3-619C3 and 3T3-SCRD3 cells. Western blotting analysis of ATG7, phospho-ERK1/2, caspase-3 cleavage, GRP78/Bip, CHOP (**A**) and p62 (**B**) in cells cultured overnight with H_2_O_2_ (10 µM) and treated with metformin (10 mM) for 24 (**A**,**B**) and 48 (**B**) hours. (**C**) Western blotting analysis of caspase-3 cleavage in 3T3-SCRD3 cells treated with cisplatin and/or zVAD-fmk for 48 hours. Total ERK1/2 (**A**) and actin (**A,B**,**C**) were used as loading control. *N* = 3; Cropped blots are shown; Uncropped blots are presented in Supplementary Fig. S3. (**D**) Gene expression analysis of shATG7 and scrambled transfected NIH/3T3 cells cultured for 24 hours with H_2_O_2_ (10 µM) and treated with metformin (10 mM) for 4 hours. Data are means ± SD. *N* = 3; *P < 0.05, **P < 0.01, ***P < 0.001.

To evaluate the molecular mechanisms involved in metformin-induced autophagic cell death, apoptosis markers and MAP kinases activation were analyzed by western blotting analysis. 3T3-619C3 and 3T3-SCRD3 cells were treated with 10 µM H_2_O_2_ for 24 hours to induce cell damage, and then treated with metformin for additional 24 hours. Metformin was able to inhibit ERK1/2 phosphorylation in 3T3-SCRD3 cells, but not in shATG7 3T3-619C3 cells (Fig. 5A). To evaluate the induction of the autophagic flux by metformin, p62 accumulation was evaluated. The results showed that metformin induced an increase in p62 expression after 24 hours, and a decrease after 48 hours in 3T3-SCRD3 cells; this effect was not revealed in shATG7 3T3-619C3 cells (Fig. 5B), indicating that autophagy was efficiently inhibited in the ATG7-silenced cells.

On the contrary, results did not reveal caspase-3 cleavage (Fig. 5A), suggesting that metformin did not inhibit cell viability via apoptosis. Again, as positive control of apoptosis, 3T3-SCRD3 cells were treated with cisplatin and zVAD-fmk. Our results show that cisplatin induced a dose-dependent caspase-3 cleavage after 48 hours, that was found to be inhibited in zVAD-fmk-treated cells (Fig. 5C). These results suggest that metformin could induce autophagic cell death inhibiting molecular pathways involved in cell proliferation and/or survival.

We also analyzed the UPR markers in cells treated with H_2_O_2_, metformin or combinations by qPCR and western blotting. The 3T3-619C3 and 3T3-SCRD3 were treated with 10 µM H_2_O_2_ for 24 hours, and then treated with metformin for additional 4 hours. Our results showed that metformin significantly induced the overexpression of *CHOP*, *ATF4*, *ATF3*, *CHAC1* and *CEBPB* genes in 3T3-619C3, while only *ATF3* and *CHAC1* were found to be significantly upregulated in 3T3-SCRD3, but to a lower extent than in 3T3-619C3 cells (Fig. 5D). In H_2_O_2_-pretreated 3T3-619C3 cells, *CHOP*, *ATF4*, *ATF3* and *CHAC1* genes were found to be upregulated by metformin (Fig. 5D). However, the expression levels of both spliced and unspliced XBP-1 and PDI mRNAs did not increase neither in 3T3-619C3 nor in 3T3-SCRD3 cells (Fig. 5D). Western blotting analysis confirmed the overexpression of CHOP in 3T3-619C3 treated with metformin after H_2_O_2_ pretreatment, and, interestingly, also revealed an overexpression of GRP78/Bip in metformin-treated cells, differently to the gene expression analysis in which GRP78/Bip was found significantly upregulated in H_2_O_2_-treated cells only (Fig. 5A). Taken together, our results showed that the UPR was preferentially induced by metformin in damaged, autophagy-defective cells.

## Discussion

Given that cancer is a complex multistage process and that autophagy performs its effects in different ways, the role of autophagy in tumorigenesis can be evaluated in a context-dependent manner. Once cancer occurs, autophagy is upregulated to make the survival of cancer cells in the hostile tumor microenvironment. On the other hand, it can be considered as a cytoprotective pathway, that prevents chronic tissue damage which could lead to cancer initiation and progression; in this case, autophagy stimulation or restoration could be useful for chemoprevention.

Metformin, a biguanide anti-diabetic drug, is able to trigger autophagy by AMPK activation and subsequent inhibition of mTOR, which is one of major inhibitor of the autophagic flux^14^. Epidemiological studies showed that the use of metformin in diabetic patients is associated with a decrease in various types of cancer incidence, most significantly in pancreatic cancer, hepatocellular carcinoma, and colon cancer^12,20,21^. However, whether metformin has activity against cancer in non-diabetics still has to be demonstrated. It has been hypothesized that metformin could act with both direct and indirect mechanisms, primarily decreasing glucose, IGF-1 and insulin signaling, thereby creating an unfavorable environment for tumor growth that is similar to that created by caloric restriction^22,23^. Through AMPK activation and mTORC signaling inhibition, metformin suppresses protein synthesis and cell proliferation^24,25^.

In this study, we evaluated the effect of metformin on tumor cell progression; for this purpose, the preneoplastic cell line JB6 cl 41-5a P+ has been used. The JB6 P+ cells are sensitive to TPA stimulation, which induces growth in soft agar and foci formation in adherent culture conditions, two hallmarks of tumorigenesis. Our results show a cytostatic effect of metformin in JB6 P+ cells. However, in presence of TPA, metformin induces massive cell death in both anchorage dependent and independent culture conditions. In adherent cells, the cytotoxic effect of metformin was inhibited by wortmannin but did not change using the pan-caspase inhibitor z-VAD-fmk, indicating an autophagy-related effect and excluding apoptosis. The autophagic process may be essential for cell death in certain settings, in which excessive or uncontrolled levels of autophagy are able to induce autophagy-dependent cell death^26^. These results underline the potential of metformin as antitumor agent: we can suppose a selective effect of this molecule, that eliminate only preneoplastic cells stimulated to form a tumor mass. At molecular level, we found an inhibition of ERK1/2 and JNK phosphorylation only in cells treated with metformin and TPA, but not in presence of wortmannin, indicating that metformin could act via autophagy in the inhibition of proliferative-related signaling.

Autophagy is a process that involves several proteins, starting from phagophores formation and multimerization of Atg proteins, to LC3 processing and insertion in extending phagophores membrane, targets capturing for degradation and authophagosomes – lysosomes fusion^27^. ATG7 is a protein with a pivotal role for the initial step of autophagy, being essential during the formation of phagophores: it acts like an E1 ubiquitin activating enzyme, activating ATG5-ATG12 conjugation, which is involved in phagophore extension step^27^. Loss of autophagy through *Atg7* knockdown causes severe physiological dysfunction; for instance, *Atg7*-defective mice showed neurodegeneration^28^ and undergone to atrophy, myopathy and loss of muscle mass^29,30^.

Our results show that the silencing of the *Atg7* gene leads to an increased cell transformation of the non-tumorigenic NIH/3T3 cells induced by tumor promoters. In fact, after two-stage cell transformation with MNU and H_2_O_2_, only shATG7 cells (3T3-619C3 cells) showed a significant increase of anchorage-independent growth and foci formation in anchorage-dependent culture conditions.

Despite it has been shown that loss of *Atg7* is not sufficient to trigger a tumor-prone phenotype^4^, our results suggest that ATG7 could have a role in the maintenance of cell integrity under stress conditions, eliminating damaged proteins and organelles that could induce tumorigenesis, and supporting the role of autophagy induction for cancer prevention.

Autophagic pathways are strictly related to ER stress. The ER is responsible for post-translational modifications and appropriate folding of proteins. Because this process is complex and error-prone, the capacity of ER can be saturated under some physiological or pathological conditions, such as glucose deprivation, hypoxia, oxidative injury or increased protein synthesis. In these cases, accumulation of unfolded proteins occurs and leads to a signaling response, the UPR, which is activated to recovery ER functions, through translation attenuation, upregulation of the folding machinery, and ER-associated degradation mediated by ubiquitin-proteasome complex and autophagy^18^. Therefore, autophagy represents a fundamental mechanism that targets both partially processed proteins and protein aggregates^31^.

In this context, we found a persistent ER stress condition in autophagy-deficient cells, revealed by UPR markers upregulation (CHOP, ATF3, ATF4, GRP78/BiP, CHAC1 and CEBPB) in 3T3-619C3 cells treated with H_2_O_2_, suggesting an accumulation of damaged proteins and corroborating the autophagy-UPR crosstalk. These results underline the importance to inhibit damaged protein accumulation for carcinogenesis prevention.

Despite autophagy plays a crucial pro-survival role in cell homeostasis, it has been shown that in extreme conditions of starvation or stress, autophagy could induce a programmed cell death^32,33^.

We analyzed the effect of metformin in tumor initiation using the NIH/3T3 cells and the ATG7-silenced NIH/3T3 clones. This cell line is characterized by a high proliferation rate, making difficult to distinguish between antiproliferative or cytotoxic effect. However, we analyzed the effect of autophagy induction using metformin in our cell models. Our results show that under stress conditions induced by MNU/H_2_O_2_, the effect of metformin in NIH/3T3 cells seems to be cytotoxic. Interestingly, this effect was not revealed in 3T3-619C3 cells, suggesting that metformin is able to induce autophagy-related cell death in damaged cells, eliminating potential tumorigenic cells. These evidence need to be deeply analyzed, improving cell damage accumulation in order to treat cells for shorten time, reducing cell proliferation.

The mitogen-activated protein kinases (MAPKs) signaling pathway is a key regulator of cell growth and survival in physiological and pathological processes and has a critical role in driving tumor initiation and progression^34^. We revealed a correlation between metformin effect and the inhibition of ERK1/2 phosphorylation, which was found to be inhibited only in 3T3-SCRD3 cells. This suggests that metformin reduces cell proliferation and survival through autophagy, inhibiting ERK1/2 signaling.

The UPR inhibition is another mechanism that has been proposed for the potential antitumorigenic effect of metformin^14^. Under stress conditions such as in solid malignancies, cells increase the protein folding capacity of the ER; if this is inhibited, cells activate signaling pathways leading to cell death, as demonstrated in human cancer cells treated with metformin^17^. In addition, the activation of UPR branches has been widely reported in a variety of human tumors including glioblastoma, lymphoma, myeloma and carcinoma of the cervix and breast^19^. The UPR is mediated by 3 transmembrane ER stress sensors IRE1, PERK and ATF6, which activate 3 signaling cascades to restore ER homeostasis. The 3 UPR branches and autophagy are strictly interconnected^18^. In fact, the IRE1 signaling can regulate autophagy through induction of sXBP1; ATF6, which induces XBP1 expression, can cooperate with IRE1; the PERK signaling can promote autophagy through induction of SESN2 and DDIT4/REDD1, two inhibitors of mTOR^18^.

In autophagy-deficient cells (3T3-619C3), the treatment with metformin significantly activated the PERK/eIF2α/ATF4 branch of UPR, compared to 3T3-SCRD3 cells, as revealed by the upregulation of PERK signaling UPR downstream markers (CHOP, ATF3, ATF4, GRP78/BiP, CHAC1 and CEBPB).

On the other hand, the IRE1 pathway appeared not to be activated in both cell lines, since the splicing induction of XBP-1 was not detected. Furthermore, the activation of the ATF-6 was not observed since the expression levels of XBP-1^35^ and PDI mRNAs (two ATF6 target genes) did not increase in metformin-treated cells. Our results revealed an increase of UPR markers in 3T3-619C3 cells treated with metformin, especially with the addition of H_2_O_2_, suggesting that metformin could mitigate ER stress by inducing autophagy. In this view, the accumulation of unfolded/damaged proteins, that cannot be eliminated by the autophagic flux, results in an upregulation of UPR.

**Figure 6:**
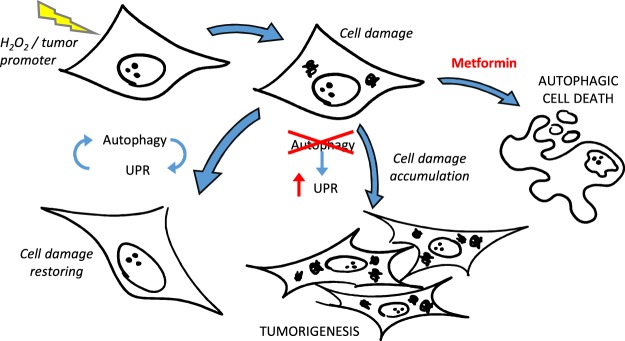
Representative scheme of results. Cells exposed to stressor or tumor promoter undergo cell damage; the autophagy-UPR crosstalk restores damaged protein and organelle maintaining cell homeostasis; inhibiting autophagy, the UPR increased indicating an accumulation of damaged proteins, which could induce tumorigenesis; metformin could trigger autophagic cell death in damaged cells eliminating potentially tumorigenic cells.

In conclusion (Fig. 6), the autophagy-UPR crosstalk could protect cells exposed to stress or tumor promoter, preventing the accumulation of damaged proteins and organelles and maintaining cell homeostasis; if autophagy is inhibited, cell damage could accumulate inducing tumorigenesis; metformin could inhibit this process inducing a selective autophagic cell death, *de facto* preventing tumor initiation. Although preliminary, these findings support the epidemiological evidence of metformin chemopreventive properties, and provide the rationale to deeply analyze the role of autophagy inducers in foods^36^ and physical exercise-induced autophagy^37^ for cancer prevention.

## Materials and Methods

### Cell lines and cell culture

The JB6 Cl 41-5a promotion-sensitive (JB6 P+) cell line was obtained from the American Type Culture Collection (ATCC, Rockville, MD, USA); the NIH/3T3 cell line was kindly provided by Dr. Stefano Amatori (European Institute of Oncology, Milan - Italy) and verified in our laboratory. Cells were cultured in DMEM (NIH/3T3) or EMEM (JB6 P+) media supplemented with 10% or 5% fetal bovine serum (FBS) for NIH/3T3 or JB6 P+ cells respectively, and 2 mmol/L L-glutamine, 1 × MEM Non-essential Amino Acid Solution, 0.1 mg/ml streptomycin, 0.1 U/L penicillin and 1 mM Na-pyruvate (JB6 P+ only). Cells were maintained in a humidified incubator (5% CO_2_) at 37 °C during at maximum fifteen passages. All cell culture materials were purchased from Sigma-Aldrich (St. Louis, MO, USA).

### ATG7 silencing and clones isolation

Stable transfection in NIH/3T3 cells was carried out using TransIT®-X2 (Mirus Bio, Madison, WI, USA) as transfection reagent according the manufacturer’s instructions. Cells were plated in 12-well plates at a density of 1 × 10^5^ cells/dish in antibiotics free culture media, incubated overnight and transfected with shRNA plasmid panels (OriGene Technologies, Inc., Rockville, MD, USA) containing four ATG7-specific shRNA sequences (Supplementary Materials, Table S1) and one non-effective scrambled shRNA sequence. After 72 hours, cells were harvested and plated in 60 mm dishes at a density of 1 × 10^5^ cells/dish with 1 µg/ml puromycin for the antibiotic selection step. After 1 week, cells were harvested and plated in 96-well plates at a density of 1 cell/well with 1 µg/ml puromycin. Next, single cell clones were expanded in 24-well plates and analyzed in western blotting for ATG7 expression. ATG7-silenced clones were stored in liquid nitrogen vapor phase for further experiments.

### Anchorage-independent transformation assay (soft-agar assay)

Wild type NIH/3T3 cells, ATG7-silenced clone 3T3-619C3 and scrambled transfected 3T3-SCRD3 were seeded on 35 mm cell culture dishes at a density of 2 × 10^4^ cells/dish. After overnight incubation, cells were treated for 1 hour with *N*-methyl-*N*-nitrosourea (MNU), washed with fresh medium and cultured in growth medium containing 10 µM H_2_O_2_. Weekly, cells were harvested and passaged at a density of 2 × 10^4^ cells/dish in growth medium containing 10 µM H_2_O_2_. The passages were repeated up to three times. At passage two and three, 5 × 10^4^ cells/35-mm dish of each condition were seeded in soft agar as described previously^38^. Briefly, cells were suspended in 1 ml of 0.3% w/v agar in complete medium, and layered over the bottom layer consisted of 1 ml of 0.6% w/v agar in complete medium.

JB6 P+ cells were directly suspended in 0.3% agar in complete medium with 10 ng/ml of 12-O-Tetradecanoylphorbol 13-acetate (TPA), 10 mM metformin, 2 μM wortmannin or combinations, and layered over the bottom layer.

Weekly, 500 μl of complete medium was added to maintain humidity. Cells were incubated at 37 °C and 5% of CO_2_ for 21 days. The colonies were then stained with crystal violet (0.01% w/v) and counted with a stereoscope. Only clusters containing more than 20 cells were counted as colonies.

### Cell viability assay

Wild type NIH/3T3 cells, ATG7-silenced clones 3T3-619C3, 3T3-619D3, 3T3-621B1, 3T3-621B6, 3T3-430A1 and scrambled transfected 3T3-SCRD3 cells were seeded at a density of 2 × 10^4^ cells/dish in 35 mm dishes, and treated as indicated in the figures. After 6 days, cells were trypsinized, plated at a density of 2 × 10^4^ cells/dish in 35 mm dishes, and treated for additional 6 days. Cell viability was evaluated by trypsinization and cell counting by trypan blue exclusion assay using a hemocytometer.

### Western blotting

After treatments, cellular protein expression and phosphorylation were analyzed by western blotting as previously reported^39^. Briefly, cells were lysed for 20 min on ice with 20 mmol/L HEPES (pH 7.9), 25% v/v glycerol, 0.42 mol/L NaCl, 0.2 mmol/L EDTA, 1.5 mmol/L MgCl2, 0.5%v/v Nonidet P-40, 1 mmol/L NaF, 1 mmol/L Na3VO4, and 1× complete protease inhibitor cocktail (Roche Diagnostics Ltd., Mannheim, Germany). The cell lysates were frozen and thawed twice and clarified by centrifugation at 12,000 rpm for 10 min at 4 °C. Total cell lysates were fractionated by SDS-PAGE and transferred to a nitrocellulose membranes (0.2 μm pore size) (Bio-Rad Laboratories Inc., Hercules, CA, USA). The following primary antibodies have been used: ATG7 (#2631), LC3B (#2775), phospho-p38 MAPK (Thr180/Tyr182) (#9211), p38 MAPK (#9212), phospho-SAPK/JNK (Thr183/Tyr185) (#9251), SAPK/JNK (#9252), phospho-p44/42 MAPK (ERK1/2) (Thr202/Tyr204) (#9101), p44/42 MAPK (ERK1/2) (9102), BiP (C50B12) (#3177), CHOP (L63F7) (#2895) and Caspase-3 (#9662) purchased from Cell Signaling Technology (Beverly, MA, USA); Actin (#A2066) purchased from Sigma-Aldrich. Protein bands were detected using a horseradish peroxidase-conjugated secondary antibody (Bio-Rad Laboratories Inc). Blots were treated with enhanced chemiluminescence reagents and the immunoreactive bands were detected with chemiluminescence film (Amersham Hyperfilm ECL; GE Healthcare, Little Chalfont, UK).

### Immunofluorescence

The NIH/3T3, 3T3-619C3 and 3T3-SCRD3 cells were seeded in 4-well chamber slide at a density of 5 × 10^4^ cells/well, incubated overnight and serum starved for 4 hours. Next, cells were fixed with 4% paraformaldehyde for 15 minutes, permeabilized with 0.2% TritonX-100, blocked with 1% of goat serum and incubated overnight at 4 °C with LC3A/B (D3U4C) (Alexa Fluor 488 Conjugate; #12082; Cell Signaling). After DAPI staining, cells were mounted with Fluoreshield (Sigma) and photographed with a fluorescente microscope.

### Quantitative real-time PCR (qPCR)

The NIH/3T3, 3T3-619C3 and 3T3-SCRD3 cells were lysed with 700 µl QIAzol Lysis Reagent (Qiagen, Hilden, Germany) and total RNA was purified using the miRNeasy Mini kit (Qiagen), following manufacturer’s instructions. The RNA was quantified using a NanoVue Plus™ spectrophotometer (GE Healthcare Life Sciences, Piscataway, NJ, USA) and 500 ng were used for the cDNA synthesis with the PrimeScript™ RT Master Mix (Perfect Real Time) (Takara Bio Inc., Otsu, Shiga, Japan).

The expression of selected ER stress marker genes was monitored by qPCR as previously described^40^, using primers listed in Table 1. B2M (beta-2-microglobulin) and GAPDH (Glyceraldehyde 3-phosphate dehydrogenase) were used as reference genes. Differences in mRNA expression was determined using the 2^−ΔΔCt^ method^41^. All RT-qPCR expression values were the result of 3 independent biological replicates.

**Table 1:** qPCR primers used to monitor expression of ER stress-related genes.

Target mRNA	Accession number	Forward primer (5′-3′)	Reverse primer (5′-3′)
CHOP	NM_007837	GAGTCCCTGCCTTTCACCTT	TTCCTCTTCGTTTCCTGGGG
HSPA5	NM_001163434	TCCGGCGTGAGGTAGAAAAG	GGCTTCATGGTAGAGCGGAA
ATF3	NM_007498	CTCTCACCTCCTGGGTCACT	TCTGGATGGCGAATCTCAGC
ATF4	NM_001287180	GCAGTGTTGCTGTAACGGAC	ATCTCGGTCATGTTGTGGGG
CEBPB	NM_009883	ACCGGGTTTCGGGACTTGA	TTGCGTCAGTCCCGTGTCCA
CHAC1	NM_026929	TATAGTGACAGCCGTGTGGG	GCTCCCCTCGAACTTGGTAT
SXBP1	NM_001271730	CTGAGTCCGCAGCAGGT	TGTCCAGAATGCCCAAAAGG
UXBP1	NM_013842	CCGCAGCACTCAGACTATG	TGTCCAGAATGCCCAAAAGG
PDI	NM_011032.3	GATCAAGCCCCACCTGATGA	ACCTCTTCAAAGTTCGCCCC
Β2M	NM_009735	TGCTATCCAGAAAACCCCTCAA	GGATTTCAATGTGAGGCGGG
GAPDH	NM_001289726	TGCCCCCATGTTTGTGATG	TGTGGTCATGAGCCCTTCC

CHOP, Mus musculus C/EBP homologous protein; HSPA5, Mus musculus heat shock protein 5; ATF3, Mus musculus activating transcription factor 3; ATF4, Mus musculus activating transcription factor 4; CEBPB, Mus musculus CCAAT/enhancer binding protein (C/EBP), beta; CHAC1, Mus musculus ChaC, cation transport regulator 1; sXBP1, Mus musculus X-Box Binding Protein 1, transcript variant 2 (spliced); uXBP1, Mus musculus X-Box Binding Protein 1, transcript variant 1 (unspliced); PDI, Mus musculus Protein disulfide-isomerase; B2M, Mus musculus Beta-2-Microglobulin; GAPDH, Mus musculus glyceraldehyde-3-phosphate dehydrogenase.

### Statistical analysis

Statistical analyses were performed using the unpaired student’s t test, and one-way or two-way ANOVA as appropriate, followed by Bonferroni’s multiple comparison post hoc tests (GraphPad Software, Inc., La Jolla, CA, USA).

## Supplementary information

Supplementary figures

## References

[1] De Flora S (2005). Overview of mechanisms of cancer chemopreventive agents. Mutat Res.

[2] Karantza-Wadsworth V (2007). Autophagy mitigates metabolic stress and genome damage in mammary tumorigenesis. Genes Dev.

[3] Mathew R (2007). Autophagy suppresses tumor progression by limiting chromosomal instability. Genes Dev.

[4] Steeves MA (2010). Targeting the autophagy pathway for cancer chemoprevention. Curr Opin Cell Biol.

[5] Chen H-Y (2011). Role of autophagy in cancer prevention. Cancer Prev Res.

[6] Koren I (2012). Promoting tumorigenesis by suppressing autophagy. Science.

[7] Pollak M (2010). Metformin and other biguanides in oncology: advancing the research agenda. Cancer Prev Res.

[8] Quinn BJ (2013). Repositioning metformin for cancer prevention and treatment. Trends Endocrin Met.

[9] Kasznicki J (2014). Metformin in cancer prevention and therapy. Ann Transl Med.

[10] Cazzaniga, M. *et al*. Breast cancer metabolism and mitochondrial activity: the possibility of chemoprevention with metformin. *Biomed Res Int* 972193 (2015). 10.1155/2015/972193 PMC4641168 26605341

[11] Heckman-Stoddard B (2016). Repurposing old drugs to chemoprevention: the case of metformin. Semin Oncol.

[12] DeCensi A (2010). Metformin and cancer risk in diabetic patients: a systematic review and meta-analysis. Cancer Prev Res.

[13] Chiang GG (2007). Targeting the mTOR signaling network in cancer. Trends Mol Med.

[14] Kourelis TV (2012). Metformin and cancer: new applications for an old drug. Med Oncol.

[15] Schroder M (2005). The mammalian unfolded protein response. Annu Rev Biochem.

[16] Boyce M (2006). Cellular response to endoplasmic reticulum stress: a matter of life or death. Cell Death Differ.

[17] Saito S (2009). Chemical genomics identifies the unfolded protein response as a target for selective cancer cell killing during glucose deprivation. Cancer Res.

[18] Rashid H-O (2015). ER stress: Autophagy induction, inhibition and selection. Autophagy.

[19] Tameire F (2015). Cell intrinsic and extrinsic activators of the unfolded protein response in cancer: Mechanisms and targets for therapy. Semin Cancer Biol.

[20] Evans JM (2005). Metformin and reduced risk of cancer in diabetic patients. BMJ.

[21] Noto H (2012). Cancer risk in diabetic patients treated with metformin: a systematic review and meta-analysis. PLoS ONE.

[22] Oliveras-Ferraros C (2010). Pharmacological mimicking of caloric restriction elicits epigenetic reprogramming of differentiated cells to stem-like self-renewal states. Rejuv Res.

[23] Anisimov VN (2010). Metformin for aging and cancer prevention. Aging.

[24] Dowling RJ (2007). Metformin inhibits mammalian target of rapamycin-dependent translation initiation in breast cancer cells. Cancer Res.

[25] Zakikhani M (2006). Metformin is an AMP kinase-dependent growth inhibitor for breast cancer cells. Cancer Res.

[26] Liu Y, Levine B (2015). Autosis and autophagic cell death: the dark side of autophagy. Cell Death Differ.

[27] Glick D (2010). Autophagy: cellular and molecular mechanisms. J Pathol.

[28] Komatsu M (2007). Essential role for autophagy protein Atg7 in the maintenance of axonal homeostasis and the prevention of axonal degeneration. P Natl Acad Sci USA.

[29] Masiero E (2009). Autophagy is required to maintain muscle mass. Cell Metab.

[30] Masiero E (2010). Autophagy inhibition induces atrophy and myopathy in adult skeletal muscles. Autophagy.

[31] Houck SA (2014). Quality control autophagy degrades soluble ERAD-resistant conformers of the misfolded membrane protein GnRHR. Mol Cell.

[32] Ouyang L (2012). Programmed cell death pathways in cancer: a review of apoptosis, autophagy and programmed necrosis. Cell Prolif.

[33] Mizushima N (2008). Autophagy fights disease through cellular self-digestion. Nature.

[34] Low HB (2016). Regulatory roles of MAPK phosphatases in cancer. Immune Network.

[35] Yoshida H (2001). XBP1 mRNA is induced by ATF6 and spliced by IRE1 in response to ER stress to produce a highly active transcription factor. Cell.

[36] Singletary K (2008). Diet, autophagy, and cancer: a review. Cancer Epidem Biomar.

[37] Koelwyn GJ (2017). Exercise-dependent regulation of the tumour microenvironment. Nat Rev Cancer.

[38] Schiavano GF (2015). Inhibition of breast cancer cell proliferation and *in vitro* tumorigenesis by a new red apple cultivar. PLoS ONE.

[39] De Santi M (2016). Human IGF1 pro-forms induce breast cancer cell proliferation via the IGF1 receptor. Cell Oncol.

[40] Galluzzi L (2016). Leishmania infantum induces mild unfolded protein response in infected macrophages. PLoS ONE.

[41] Pfaffl MW (2001). A new mathematical model for relative quantification in real-time RT–PCR. Nucleic Acids Res.

