# Supplementary material for: Metformin prevents cell tumorigenesis through autophagy-related cell death
Source: Sci Rep. 2019 Jan 11;9:66. doi: 10.1038/s41598-018-37247-6 (PMC6329809; doi:10.1038/s41598-018-37247-6)

Supplementary Information

Metformin prevents cell tumorigenesis through autophagy-related cell death

Mauro De Santi [1]*, Giulia Baldelli [1], Aurora Diotallevi [2], Luca Galluzzi [2], Giuditta Fiorella Schiavano [1], Giorgio Brandi [1]

Authors' affiliation

[1] Department of Biomolecular Sciences, Hygiene Unit, University of Urbino Carlo Bo, Urbino (PU), Italy.

[2] Department of Biomolecular Sciences, Biotechnology Unit, University of Urbino Carlo Bo, Urbino (PU), Italy.

*** Corresponding Author:**

Mauro De Santi

Department of Biomolecular Sciences, Hygiene Unit, University of Urbino Carlo Bo;

Via S. Chiara, 27 – 61029 Urbino (PU) ITALY

Email: mauro.desanti@uniurb.it

Supplementary Figure S1 - full-length blots of figure 2

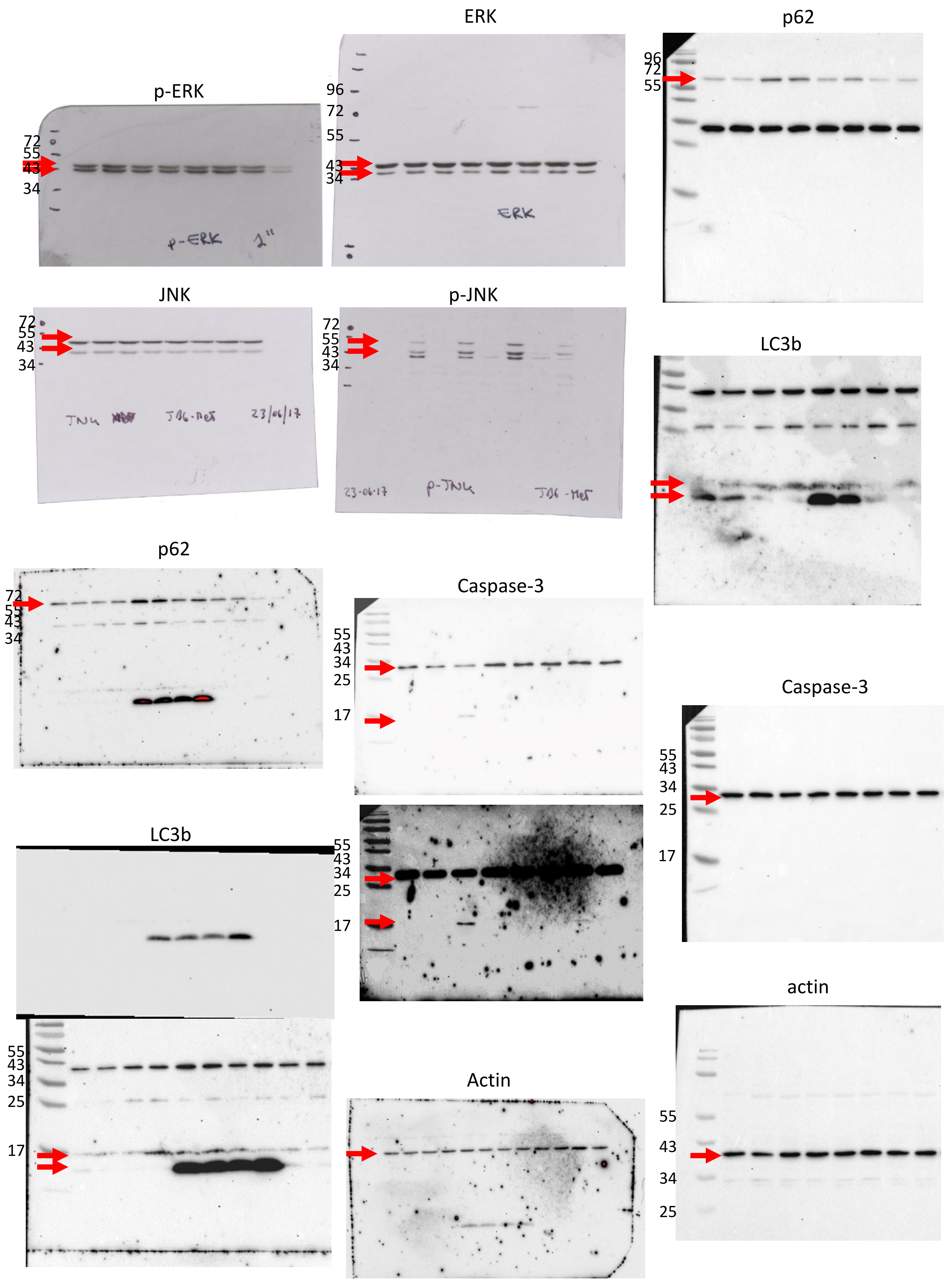

Supplementary Figure S2 - full-length blots of figure 3

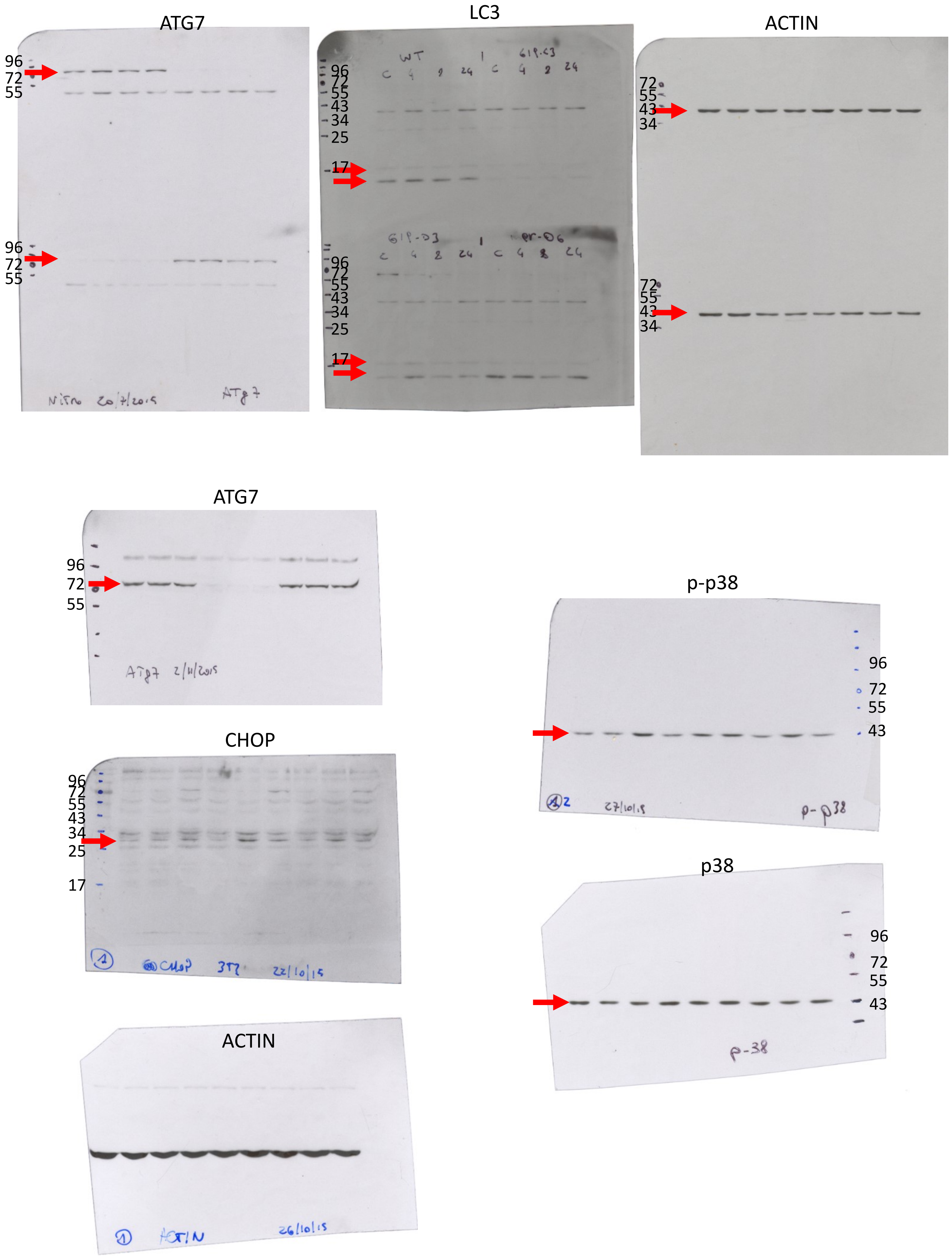

Supplementary Figure S3 - full-length blots of figure 5

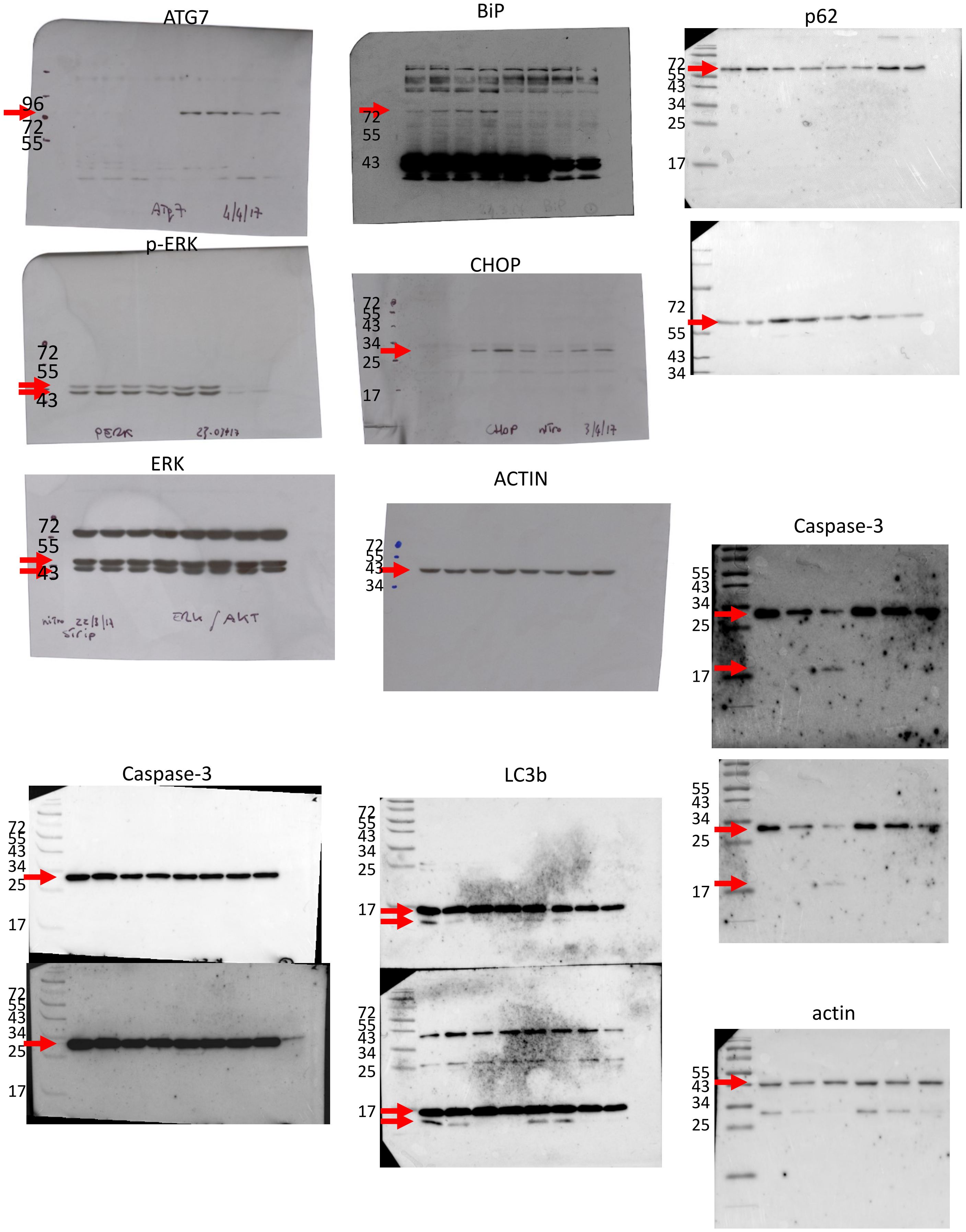

Supplement: Supplementary file 1 — Supplementary figures [file 41598_2018_37247_MOESM1_ESM.pdf]
